# Thalamo-cortical cross-frequency coupling detected with MEG

**DOI:** 10.3389/fnhum.2014.00187

**Published:** 2014-04-02

**Authors:** Bernadette C. M. van Wijk, Thomas H. B. FitzGerald

**Affiliations:** Wellcome Trust Centre for Neuroimaging, Institute of Neurology, University College LondonLondon, UK

**Keywords:** magnetoencephalography, phase-amplitude coupling, alpha band, gamma band, thalamo-cortical interactions, source space analysis, cross-frequency coupling

Neural oscillations are observed across a variety of temporal and spatial scales, and are believed to play a key role in brain function. In addition to specific functional roles ascribed to isolated frequencies, it is likely that the interplay between activity at different frequencies plays an essential role in cognition and behavior (Canolty and Knight, 2010). As such, cross-frequency interactions have become the focus of a large literature exploring different types of coupling across a multitude of brain regions and states (Jirsa and Müller, 2013). Coupling between low and high frequency signal components may occur in different forms involving either the phase, amplitude or frequency of the signals. The best studied form is phase-amplitude coupling, in which the amplitude of higher frequency activity is modulated with the phase of activity in a lower frequency band. Most work to date has, however, focused on local cross-frequency interactions, leaving coupling between brain regions relatively little explored. These non-local interactions are interesting because they provide a means by which neuronal activity can be coordinated across both space and time. The paper by Roux et al. (2013) stands out because it addresses this issue non-invasively in humans.

Using magnetoencephalography (MEG), Roux et al. (2013) analyzed data collected from 45 healthy participants during quiet wakefulness with eyes shut. Time series for left and right thalamus, and all other cortical and subcortical locations, were extracted by projecting band-pass filtered data through beamformer spatial filters. The instantaneous phase of thalamic alpha band activity (8–13 Hz) and the instantaneous amplitude of cortical gamma activity (30–70) were used to test for phase-amplitude coupling by computing the modulation index (Tort et al., 2008). This revealed a significant thalamo-cortical cross-frequency coupling with the posterior medial parietal cortex (PMPC), one of the hubs in the default mode network.

These results are exciting, but raise an obvious concern. The thalamus is deeply located close to the center of the head and it can, therefore, be doubted whether its signal-to-noise ratio is sufficient for reliable alpha phase estimation. Furthermore, spurious thalamo-cortical coupling might have been introduced by leakage from sources elsewhere in the brain. It is vital to rule this out, and in general assess the plausibility of estimating thalamo-cortical coupling using MEG. Recently, Attal and Schwartz (2013) showed that significant thalamic sources could be detected when contrasting alpha power between eyes open and eyes closed. In addition, thalamo-cortical alpha band synchronization has been reported previously (Pollok et al., 2005). The *prima facie* plausibility of cross-frequency coupling between subcortical structures and the cortex on the other hand, is demonstrated by studies reporting thalamo-cortical phase-amplitude coupling using data collected invasively from the thalamus (Fitzgerald et al., 2013) and subthalamic nucleus (de Hemptinne et al., 2013). Taken together, existing literature provides some support for both the approach pursued by Roux et al. (2013) and the findings they present, however without obviating the need for careful supplementary analysis to support their findings.

To this end, the authors report three extra analyses. Firstly, the transfer entropy between the raw signals from thalamus and cortex was estimated at different time lags. This indicated that information flow was directed from thalamus to cortex, and was maximal at a lag in line with physiologically plausible conduction delays. Secondly, to verify that coupling was specific to the thalamus, the authors took the gamma amplitude at PMPC as a seed and tested for coupling with the phase at all other source locations. Significant coupling was confined to the thalamus and did not appear in other regions except that gamma amplitude was also significantly coupled to alpha phase within the PMPC. Finally, correlations between spatial filters at the thalamus and all other locations in the brain supported the absence of leakage from signals at cortical locations into those of the thalamus.

These analyses mitigate concerns that thalamo-cortical coupling resulted from volume conduction artefacts although further analyses could have been performed to establish this more definitively. In particular, as the authors also report phase locking between the thalamus and PMPC within the alpha band, a non-zero phase delay for this locking would argue against volume conduction. An alternative approach would have been to orthogonalize the thalamic and cortical time series as demonstrated by Hipp et al. (2012). Here, before analysing dependencies between two signals all zero-phase correlated activity is regressed out of one signal, ensuring that the time series no longer share any activity picked up from common sources.

From a broader perspective, a key question is how best to understand different types of cross-frequency coupling functionally and neurophysiologically. One possibility is that activity at the frequencies of interest plays clearly defined functional roles as a pair of oscillations, such as segregating and maintaining neuronal representations of different items in working memory (Lisman and Jensen, 2013). However, an equally plausible alternative is that cross-frequency coupling is a data feature that arises as an epiphenomenon due to particular neuronal firing patterns without functional significance in itself. This would not decrease the interest of results like those reported by Roux et al. (2013), but simply change their interpretation.

This discussion motivates the use of neurobiologically plausible models as a means to understand the generative mechanisms behind observed activity. Such models could include a detailed description of the neural dynamics of the different layers within a cortical column (Bastos et al., 2012). Cross-frequency phenomena might emerge by linking the layer-specific intrinsic firing frequencies (Spaak et al., 2012). It may turn out to be possible to explain complex changes in cross-frequency coupling patterns by adjusting a single parameter in the underlying neuronal model. This approach could also, in principle, support deductions about the causal relationships between sources underlying the observed coupling.

Understanding interactions between neuronal dynamics at different spatial and temporal scales, both within and between regions, is likely to be critical for characterizing normal and pathological brain function. The paper of Roux et al. (2013) represents a step toward this, and suggests that MEG can play a useful role in characterizing cross-frequency coupling between deep sources and the neocortex.
